# Impact of Preoperative Anaemia and Blood Transfusion on Postoperative Outcomes in Gynaecological Surgery

**DOI:** 10.1371/journal.pone.0130861

**Published:** 2015-07-06

**Authors:** Toby Richards, Khaled M. Musallam, Joseph Nassif, Ghina Ghazeeri, Muhieddine Seoud, Kurinchi S. Gurusamy, Faek R. Jamali

**Affiliations:** 1 Division of Surgery and Interventional Science, University College London Hospital, London, United Kingdom; 2 Department of Internal Medicine, American University of Beirut Medical Centre, Beirut, Lebanon; 3 Department of Obstetrics and Gynaecology, American University of Beirut Medical Centre, Beirut, Lebanon; 4 Department of Surgery, American University of Beirut Medical Centre, Beirut, Lebanon; German Red Cross Blood Service Frankfurt, GERMANY

## Abstract

**Objective:**

To evaluate the effect of preoperative anaemia and blood transfusion on 30-day postoperative morbidity and mortality in patients undergoing gynecological surgery.

**Study Design:**

Data were analyzed from 12,836 women undergoing operation in the American College of Surgeons National Surgical Quality Improvement Program. Outcomes measured were; 30-day postoperative mortality, composite and specific morbidities (cardiac, respiratory, central nervous system, renal, wound, sepsis, venous thrombosis, or major bleeding). Multivariate logistic regression models were performed using adjusted odds ratios (OR_adj_) to assess the independent effects of preoperative anaemia (hematocrit <36.0%) on outcomes, effect estimates were performed before and after adjustment for perioperative transfusion requirement.

**Results:**

The prevalence of preoperative anaemia was 23.9% (95%CI: 23.2–24.7). Adjusted for confounders by multivariate logistic regression; preoperative anaemia was independently and significantly associated with increased odds of 30-day mortality (OR: 2.40, 95%CI: 1.06–5.44) and composite morbidity (OR: 1.80, 95%CI: 1.45–2.24). This was reflected by significantly higher adjusted odds of almost all specific morbidities including; respiratory, central nervous system, renal, wound, sepsis, and venous thrombosis. Blood Transfusion increased the effect of preoperative anaemia on outcomes (61% of the effect on mortality and 16% of the composite morbidity).

**Conclusions:**

Preoperative anaemia is associated with adverse post-operative outcomes in women undergoing gynecological surgery. This risk associated with preoperative anaemia did not appear to be corrected by use of perioperative transfusion.

## Introduction

Preoperative anaemia is an emerging concern in patients undergoing surgery. This has been also been attributed to the increased need for perioperative transfusions in the anaemic subject [1–4], which are also independently associated with adverse postoperative outcomes even when as low as one unit of packed red blood cells (pRBC) is administered intraoperatively [5–9]. More recently, it has also been established that preoperative anaemia carries an increased risk of longer hospital stay and increased postoperative morbidity and mortality, irrespective of the need for transfusion therapy [10–18]. However, available data mostly come from large retrospective studies reporting overall outcomes for a heterogeneous mix of patients undergoing general, vascular, or orthopaedic procedures; often with older patients with concomitant morbidities [12]. Consequently, anaemia may be a marker of the older and more ‘high risk’ patient. The impact and effect of preoperative anaemia in a more homogenous and younger age group of patients undergoing surgery, less likely to have comorbidities’ is not known.

A major cause of uterine surgery in reproductive age women is uterine fibroids. Fibroids are found in up to 70–80% of women and may cause a number of symptoms requiring surgery [19]. Heavy menstrual bleeding is a common symptom and is probably the most frequent cause of anaemia in young-otherwise healthy women. Hysterectomy is the most common operation carried out in women with fibroids, followed by myomectomy, and these are more likely to be performed in women with symptomatic fibroids [20]. Similarly, anaemia may be a common complication of the disease or its treatment in women with gynaecologic malignancies, which may require surgical intervention along the course of disease management. Hence, women undergoing uterine surgery may often be anaemic preoperatively and are likely to be younger and relatively free of significant cardiovascular or respiratory comorbidities.

In order to address this research gap, we used data from the American College of Surgeons National Surgical Quality Improvement Program (ACS NSQIP) to determine the effects of preoperative anaemia on morbidity and mortality in women undergoing uterine surgery. We also evaluated if such effects are independent or mostly mediated by an increased requirement for perioperative transfusions.

## Materials and Methods

### Study design and sample

This is retrospective cohort study using data from the ACS NSQIP database. Details of the ACS NSQIP (www.acsnsqip.org) have been recently described [12] and are summarized in supplementary **S1 Table**. It is a validated outcomes registry designed to provide feedback to participating hospitals on 30-day risk-adjusted surgical mortality and morbidity [21,22]. The database includes de-identified data on demographics, perioperative variables, and 30-day postoperative outcomes for adult patients undergoing major surgery in participating nonveteran’s administration hospitals [21]. Trained surgical clinical reviewers collect patient data upon admission from the medical chart, operative log, anaesthesia record, interviews with the attending surgeon, and telephone interviews with the patient [21]. Data quality is ensured through comprehensive training of the nurse reviewers, an inter-rater reliability audit of participating sites, regular conference calls, and an annual meeting [23]. For this study, the available ACS NSQIP Participant Use Files of the years 2008 (271,368 cases from 211 sites) and 2009 (336,190 cases from 237 sites) were retrieved for all major surgeries performed at participating ACS NSQIP Medical Centres located in the US, Canada, Lebanon and the United Arab Emirates. In accordance with the American University of Beirut’s guidelines (which follow the US Code of Federal Regulations for the Protection of Human Subjects), institutional review board approval was not needed or sought for our analysis because data were collected as part of a quality assurance activity.

We included all women undergoing uterine surgery as identified by the Current Procedural Terminology (CPT) codes. These were reviewed by two authors and grouped into three categories: *myomectomy* (58140, 58145, 58146), *simple hysterectomy* (58150, 58152, 58180, 58260, 58262, 58263, 58267, 58270, 58275, 58280, 58285, 58290, 58291, 58292, 58293, 58294, 58541, 58542, 58543, 58544, 58550, 58552, 58553, 58554, 58570, 58571, 58572, 58573), and *complex hysterectomy* (58200, 58210, 58240, 58548, 58940, 58943, 58950, 58951, 58952, 58953, 58954, 58956, 58957, 58958). For patients having more than one procedure only the index case was included. We excluded patients with a missing preoperative haematocrit level (n = 527). The main analysis was conducted on 12,836 patients.

### Preoperative anaemia

Retrieved preoperative haematocrit (HCT) level reflected the last HCT measurement prior to the index operation. Some 99.4% of the HCT levels were obtained within two months of the index surgery, 97.5% were obtained within one month and 90.3% were obtained within two weeks. We defined preoperative anaemia as a HCT level <36.0% according to the World Health Organization’s sex-based criteria [24].

### Postoperative outcomes

Evaluated postoperative outcomes were 30-day mortality and morbidity including: (1) cardiac (acute myocardial infarction or cardiac arrest requiring cardiopulmonary resuscitation); (2) respiratory (pneumonia, ventilator support for greater than 48 hours, or unplanned intubation); (3) central nervous system (CNS) (cerebrovascular accident or coma lasting more than 24 hours); (4) renal (progressive renal insufficiency or acute renal failure); (5) wound (deep incisional surgical site infection, organ or space surgical site infection, or wound dehiscence); (6) sepsis (sepsis or septic shock); (7) venous thrombosis (deep venous thrombosis or pulmonary embolism); and (8) major bleeding (requiring transfusion of more than four pRBC units within 72 hours postoperatively) based on the definitions used by ACS NSQIP. Composite morbidity was defined as having one or more of the aforementioned major morbidities.

### Statistical analysis

Bivariate comparisons between the preoperative anaemia and no anaemia groups were done using the chi-square test for categorical variables and the independent samples *t-*test for continuous variables.

The primary study outcome measure was death within 30 days of the index surgery in the preoperative anaemia compared with the no preoperative anaemia group. The secondary study outcome measure was occurrence of morbidity (composite and specific morbidities) within 30 days of the index surgery in the preoperative anaemia compared with the no preoperative anaemia group. Separate multivariate logistic regression models for 30-day mortality, composite morbidity, and each specific morbidity were performed using adjusted odds ratios (OR_adj_). Models were built by adjusting the determinant variable (preoperative anaemia vs. no anaemia) to *a priori* defined potential confounders of clinical relevance (risk factors that may have caused both preoperative anaemia as well as adverse 30-day postoperative outcomes) including; cancer, bleeding disorder, cardiac disease diabetes etc. (Table 1). Two levels of adjustment were used, Model 1 (OR_adj-1_) with basic adjustment for the most clinically relevant variables and Model 2 (OR_adj-2_) with extended adjustment for a larger number of clinically relevant risk factors. Data were near complete, with the exception of missing values for body mass index (n = 83, 0.7%), which were imputed by the respective means of similar age groups.

**Table 1:** Patients’ characteristics.

Parameter	No preoperative anaemia (n = 9,765)	Preoperative anaemia (n = 3,071)	p-value
Age in years, mean (SD)	49.0 (12.3)	47.1 (11.3)	<0.001
Race, %			<0.001
White	76.7	58.5	
Black or African American	9.9	26.7	
Other/Unknown	13.5	14.9	
Uterine surgery, %			<0.001
Myomectomy	3.1	5.2	
Simple hysterectomy	88.4	83.5	
Complex hysterectomy	8.5	11.3	
Emergency case, %	0.6	2.8	<0.001
Diabetic on oral agents or insulin, %	7.1	8.5	0.010
Obesity^a^, %	44.0	46.4	0.020
Hypertension requiring medication, %	29.0	30.1	0.214
Currently on dialysis, %	0.1	0.2	0.034
Smoker during year prior, %	20.9	15.2	<0.001
Chronic obstructive pulmonary disease, %	1.4	1.4	0.787
Congestive heart failure, %	0.1	0.4	<0.001
Coronary artery disease^b^, %	1.5	1.8	0.248
Peripheral vascular disease^c^, %	0.1	0.5	0.005
Cerebrovascular disease^d^, %	1.6	1.9	0.349
Bleeding disorder, %	0.8	2.5	<0.001
Cancer^e^, %	1.2	3.5	<0.001
Pregnancy, %	0.0	0.3	<0.001
Prior operation within 30 days, %	0.4	1.0	<0.001

^a^Body mass index ≥30 kg/m^2^.

^b^Myocardial infarction in 6 months prior, previous percutaneous coronary intervention, or previous cardiac surgery.

^c^Requiring revascularization, angioplasty, or amputation.

^d^History of transient ischaemic attack or cerebrovascular accident with or without neurologic deficit.

^e^Chemotherapy or radiotherapy in 30 days prior, disseminated cancer, or tumor involving central nervous system.

To establish whether the effects of preoperative anaemia on 30-day postoperative mortality and morbidity are mediated by an increased requirement for perioperative transfusions, we also compared the effect estimates before and after adjustment for perioperative transfusion requirement.

To determine the potential effect of the excluded 527 patients with no preoperative HCT values on the observed association between preoperative anaemia and 30-day postoperative mortality and morbidity, we carried out two separate sensitivity analyses: one including all excluded patients in the preoperative anaemia group and another including them in the no preoperative anaemia group. This would give out the two extremes of bias, in the case where absence of preoperative HCT value was non-random.

All p-values were two sided with the level of significance set at 0.05. We carried out the data management and analyses using the SAS software version 9.1 (SAS Institute Inc, Cary, NC).

## Results

Data from 12,836 patients were included in this analysis. A total of 464 (3.6%) patients underwent myomectomy, 11,193 (87.2%) had simple hysterectomy, while 1,179 (9.2%) had complex hysterectomy. The mean age was 48.6 ± 12.1 years (range: 18–90 years). Based on the pre-set (WHO) definition of anaemia, 3,071 patients had preoperative anaemia giving a prevalence of 23.9% (95% confidence interval [CI]: 23.2–24.7). Overall incidence of major comorbidities was low in this population; cancer (1.2 & 3.5%), cardiac (1.6 & 2.2%) or renal failure (0.1 & 0.2%). Anaemic patients were slightly younger (47 v 49), more likely to be non-white and less likely to be smokers (**Table 1**).

**Fig 1:**
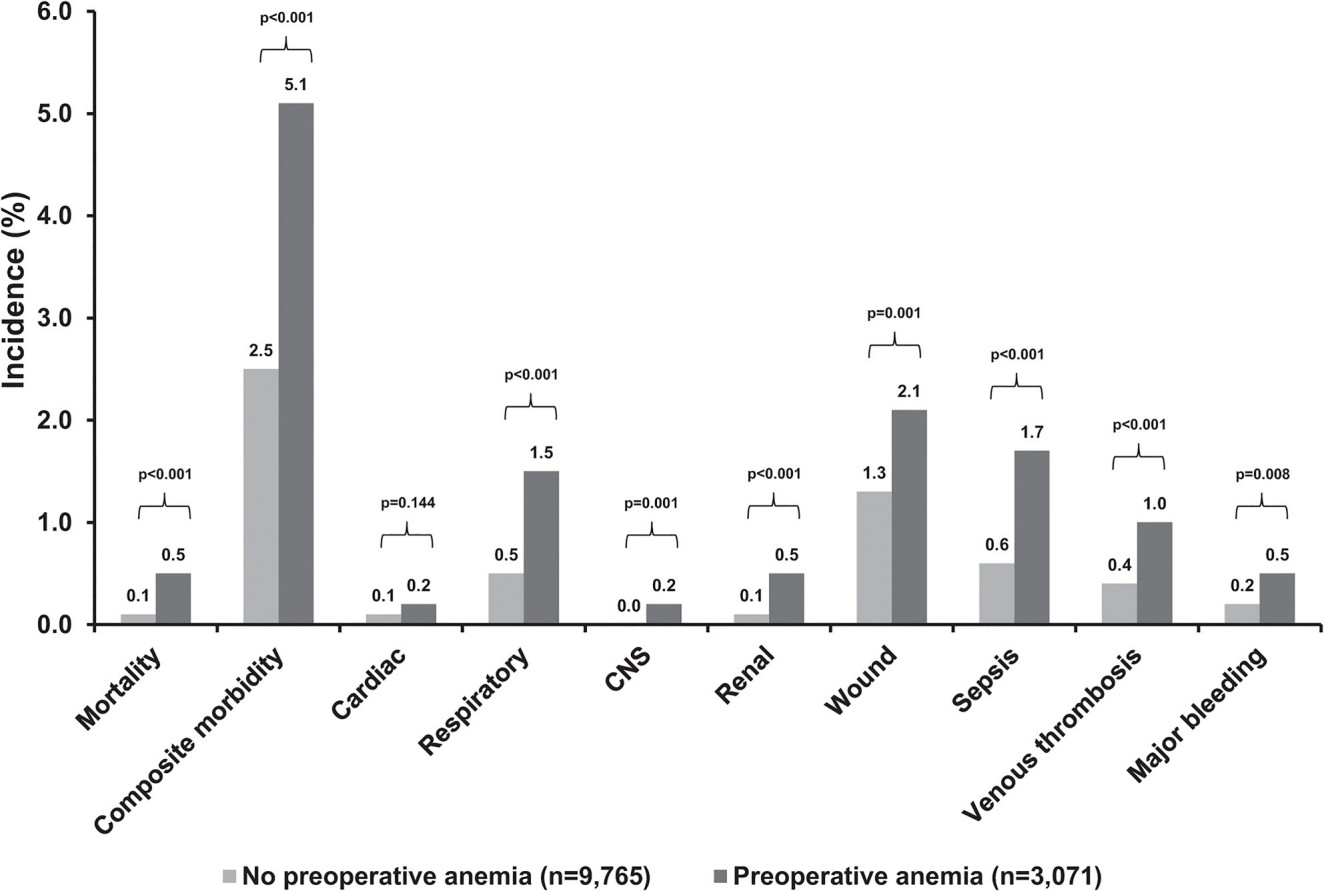
Crude 30-day postoperative mortality and morbidity rates in patients with and without preoperative anaemia. CNS, central nervous system.

**Fig 2:**
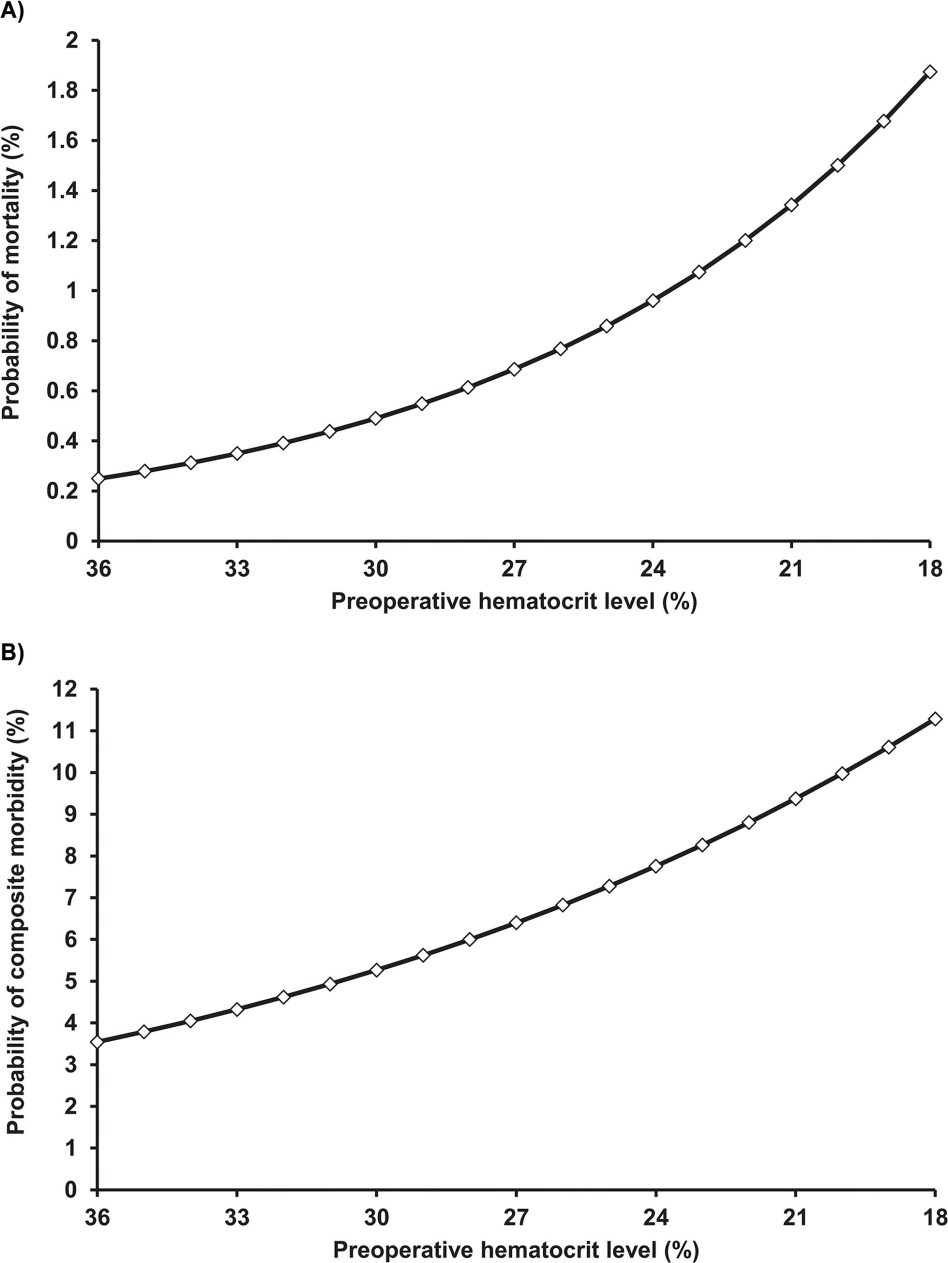
Probability of (A) mortality and (B) composite morbidity according to descending hematocrit levels in the preoperative anaemia group.

Overall patients with preoperative anaemia had a significantly higher crude 30-day mortality (0.5% vs. 0.1%, p<0.001) and composite morbidity (5.1% vs. 2.5%, p<0.001) rates than patients without preoperative anaemia (**Fig 1**). Preoperative anaemia was associated with significantly higher rates of most evaluated 30-day postoperative outcomes (**Fig 1**). Preoperative anaemia was associated with significantly higher rates probability of 30-day mortality and composite morbidity. These probabilities continued to increase with declining HCT levels in the preoperative anaemia group (**Fig 2**). After multivariate analysis adjusting for potential confounders, preoperative anaemia remained independently and significantly associated with increased 30-day mortality and morbidity (**Table 2**). The OR_adj-2_ for death was 2.40 (95% CI: 1.06–5.44) in patients with preoperative anaemia compared to patients without. The OR_adj-2_ for composite morbidity was 1.80 (95% CI: 1.45–2.24) in patients with preoperative anaemia compared to patients without. This was reflected by significantly higher adjusted odds of almost all specific morbidities including respiratory, CNS, renal, wound, sepsis, and venous thrombosis (**Table 2**). Although the adjusted odds of cardiac occurrences and major bleeding were higher in patients with preoperative anaemia compared to patients without, they had high uncertainty.

**Table 2:** Effects of preoperative anaemia on 30-day postoperative mortality and morbidity.

Outcome	Preoperative anaemia^a^		
	OR_unadj_ (95% CI)	OR_adj-1_ (95% CI)	OR_adj-2_ (95% CI)
**Mortality**	3.42 (1.65–7.09)	3.10 (1.46–6.54)	2.40 (1.06–5.44)
**Composite morbidity**	2.11 (1.72–2.59)	2.06 (1.67–2.53)	1.80 (1.45–2.24)
**Specific morbidity**			
Cardiac	2.12 (0.76–6.00)	2.21 (0.77–6.33)	2.09 (0.69–6.33)
Respiratory	3.14 (2.08–4.75)	3.12 (2.05–4.76)	2.72 (1.75–4.23)
CNS	15.92 (1.86–136.34)	13.49 (1.55–117.4)	10.45 (1.05–103.90)
Renal	6.38 (2.57–15.83)	5.56 (2.22–13.96)	5.18 (1.94–13.86)
Wound	1.63 (1.20–2.21)	1.55 (1.14–2.10)	1.44 (1.05–1.98)
Sepsis	2.70 (1.86–3.91)	2.64 (1.81–3.86)	2.15 (1.45–3.20)
Venous thrombosis	2.28 (1.43–3.66)	2.24 (1.39–3.62)	1.96 (1.19–3.23)
Major bleeding	2.48 (1.23–4.99)	2.19 (1.08–4.43)	1.27 (0.58–2.81)

^a^Patients without preoperative anaemia constituted the reference population.

OR_unadj_ = unadjusted odds ratio

OR_adj-1_ = adjusted odds ratio according to Model 1: adjusted for age, race, and type of uterine surgery.

OR_adj-2_ = adjusted odds ratio according to Model 2: adjusted for all variables in Table 1.

***Abbreviations*:** CNS, central nervous system.

In the sensitivity analyses, results remained unchanged for both the primary and secondary study outcome measures when patients with missing preoperative HCT levels (4%) were included as either having anaemia or no anaemia.

### The effect of perioperative transfusions

Patients with preoperative anaemia were more likely to receive perioperative transfusions than patients without preoperative anaemia (9.2% vs. 1.5%, p<0.001). When the association between preoperative anaemia and postoperative 30-day outcomes was further adjusted for perioperative transfusion requirement, the effect estimates (OR) dropped by around 1 point for mortality and by 0.3 points for composite morbidity (**Table 3**). In specific, the OR_adj-2_ for mortality dropped from 2.40 to 1.49 meaning that around 61% of the observed increase in 30-day mortality due to preoperative anaemia is mediated by an increased requirement for perioperative transfusion while the remaining effect is most likely attributed to an independent effect. However, the OR_adj-2_ for composite morbidity dropped from 1.80 to 1.55 meaning that only 16% of the observed increase in 30-day composite morbidity due to preoperative anaemia is mediated by an increased requirement for perioperative transfusion while the remaining effect is most likely due to an independent effect.

**Table 3:** Effects of preoperative anaemia on 30-day postoperative mortality and morbidity upon adjustment for perioperative transfusion requirement.

Outcome	Preoperative anaemia^a^	
	No adjustment for perioperative transfusion	Adjustment for perioperative transfusion
**Mortality**		
OR_unadj_ (95% CI)	3.42 (1.65–7.09)	1.90 (0.85–4.25)
OR_adj-2_ (95% CI)	3.10 (1.46–6.54)	2.13 (0.95–4.77)
OR_adj-2_ (95% CI)	2.40 (1.06–5.44)	1.49 (0.60–3.70)
**Composite morbidity**		
OR_unadj_ (95% CI)	2.11 (1.72–2.59)	1.67 (1.34–2.07)
OR_adj-2_ (95% CI)	2.06 (1.67–2.53)	1.71 (1.37–2.13)
OR_adj-2_ (95% CI)	1.80 (1.45–2.24)	1.55 (1.24–1.95)

^a^Patients without preoperative anaemia constituted the reference population.

OR_unadj_ = unadjusted odds ratio

OR_adj-1_ = adjusted odds ratio according to Model 1: adjusted for age, race, and type of uterine surgery.

OR_adj-2_ = adjusted odds ratio according to Model 2: adjusted for all variables in Table 1.

## Discussion

### Main findings

In a large series of patients these data show an independent association of preoperative anaemia with an increased risk of 30-day mortality and morbidity in females who underwent gynaecological surgery. These effects were incremental with lower haematocrit levels, and were still observed upon adjustment for the effects of patient characteristics, co-morbidities or type of uterine surgery. Blood Transfusion did not appear to ameliorate these risks and may independently be associated with increased risk of 30-day mortality and morbidity. These findings carry important clinical implications, especially since around quarter of women presenting for uterine surgery had with preoperative anaemia in this study.

### Strengths and limitations

The key strengths of our study lie in the large number of patients and the reliable and comprehensive data collection tool of the ACS NSQIP, which provides an extensive list demographic, pre-, and perioperative variables available for adjustment. These data present a homogenous area of surgery in a relatively younger healthier population with a low incidence of cardiac or respiratory disease compared to previous reports [12]. Our study carries several limitations: Uterine surgery covers various indications, the incidence of cancer was very low (<4%) and the impact of preoperative anaemia in this subgroup could not be stratified due to small numbers, the majority of patients underwent simple hysterectomy we and did not distinguish between open or laparoscopic techniques that may impact transfusion need. Approximately 2.5% of the preoperative HCT levels were obtained more than four weeks prior to surgery and may not accurately reflect the HCT levels at the time of surgery. However, variation of HCT levels in an individual is likely to be small in the absence of major bleeding, which in our database would have been identified by preoperative blood transfusions and hence considered for in the analysis looking at the effects or perioperative transfusions. Intraoperative nadir HCT or immediate postoperative HCT levels are not documented in the ACS NSQIP. Thus, we were unable to determine if lower intraoperative HCT was associated with worse outcomes.

The linkage to blood transfusion is associative. Blood transfusion is needed for blood loss replacement in a time of haemodynamic need in surgery. However, indication for blood transfusion is commonly based on HCT or haemoglobin levels rather than clinical indication and in the stable patient, restrictive transfusion practise may not be routine in all centres. NSQIP does not collect data on indication for transfusion so the associations presented cannot be fully explored. The analysis used estimates the relative effects of anaemia and transfusion on outcomes. Although causality between anaemia and transfusion is linked, the interplay with outcomes is associative. Another potential limitation of this study was that we were unable to control for hospital effects owing to the absence of hospital identifiers in our data. There may have been variability in hospital quality or variability in surgical strategy which may have potentially confounded the association between risk factors and outcome. Finally, the possibility of residual confounding is always present in observational studies such as that due to factors that we were unable to account for (e.g., socioeconomic status, malnutrition, health consciousness).

### Interpretation

Our study is in agreement with previous studies showing association of anaemia and adverse outcome in patient undergoing surgery [5–8,10,12], and shows and independent association of preoperative anaemia and adverse outcomes even in a younger and ‘fitter’ cohort of patients. The relative contribution of preoperative anaemia and perioperative transfusions on postoperative outcomes can be difficult to dissect. In our study, these data suggest that the effects of anaemia on the risk mortality and less so, on the risk of morbidity may be partly attributed to an increased requirement for transfusion use. It is not possible to link causality between either preoperative anaemia and outcomes nor transfusion and outcomes. The question of whether correction of anaemia improves these negative associative outcomes is the objective of several ongoing clinical trials in Europe [25].

Nevertheless, recognition of anaemia and transfusion practice in surgery is vitally important [2]. Patient Blood Management is an evidence-based approach to reduce risk from anaemia and blood transfusion with direct impact on surgical patient outcomes. PBM focuses on three ‘pillars’ of care in the surgical patient: the detection and treatment of preoperative anaemia; the reduction of perioperative blood loss; harnessing and optimising the patient specific physiological reserve of anaemia, including restrictive haemoglobin transfusion triggers [26–28]. Patient Blood Management has been adopted by the World Health Organisation, and recommendations have since been released for the implementation of PBM in the NHS [29].

Preoperative anaemia is readily and easily detectable and therefore a potentially preventable risk factor in patients undergoing elective surgery [30]. It is recommended that haemoglobin/haematocrit level determination is performed not less than 28 days before the scheduled elective surgical procedure, to allow for subsequent investigation and intervention in patients identified with anaemia [31]. At least, in orthopaedic [32] and cardiac [33] surgery, there is strong evidence that preoperative treatment of anaemia results in reduced intraoperative pRBC transfusion as well as improved outcome [34]. An additional approach in women due to undergo uterine surgery, is to prevent heavy menstrual bleeding by the use of gonadotropin releasing hormone analogues or ulipristal acetate [20].

### Conclusion

Preoperative anaemia in women undergoing gynaecological surgery is an independent risk for increased morbidity and mortality following surgery. Blood transfusion may not be correct this risk. Further studies are needed to assess if the management of preoperative anaemia has an impact on patient outcomes.

## Supporting Information

S1 Table The American College of Surgeons National Surgical Quality Improvement Program. (PDF)
