# Supplementary material for: Impact of Preoperative Anaemia and Blood Transfusion on Postoperative Outcomes in Gynaecological Surgery
Source: PLoS One. 2015 Jul 6;10(7):e0130861. doi: 10.1371/journal.pone.0130861 (PMC4492675; doi:10.1371/journal.pone.0130861)
Supplement: S1 Table — (PDF) [file pone.0130861.s001.pdf]

S1 Table. The American College of Surgeons National Surgical Quality Improvement Program.

Aim

The ACS NSQIP was set up as a rigorous data collection tool for outcome measurement. The ACS NSQIP collects data on a variety of clinical variables, including preoperative risk factors, intraoperative variables, and 30-day postoperative mortality and morbidity outcomes for patients undergoing major surgical procedures in both the inpatient and outpatient setting. Using validated statistical tools that were developed and tested in large population-based studies, the ACS NSQIP program generates an expected outcome based on the case complexity makes. The observed outcomes are then compared to the expected outcomes to obtain an O/E ratio and indicate performance of a particular medical centre with regards to the national average. This data is then used to identify areas in need of quality improvement.

Participants

Contribution to the ACS NSQIP is on a voluntary basis. Nonveterans hospitals that are interested in quality assurance and outcomes measurement sign-up for inclusion into the ACS NSQIP database and enter cases prospectively into this database. There are currently 289 out of 440 participating sites that are importing data to the ACS NSQIP database, located in 42 states across the US (272 sites), three Canadian provinces (15 sites), Lebanon (1 site) and the United Arab Emirates (1 site). 52% of the enrolled medical centres are classified as Academic/Teaching and 48% are Non-Teaching sites. 45% of participating medical centres have ≥500 beds, 41% have 300-499 beds, 11% have 100-299 beds, and 3% have <100 beds.

Inclusion/exclusion of cases

The ACS NSQIP includes all major surgeries as determined by CPT® codes. The ACS NSQIP has developed a comprehensive CPT® Code Inclusion List available on the website (www.acsnsqip.org). Excluded cases are:

-Patients under the age of 16 years (data collected after the year 2008 was for patients over 18 years).
-Cases listed on the CPT® Code Exclusion List on the website (www.acsnsqip.org).
-Trauma cases - specifically: A patient who is admitted to the hospital with acute trauma and has surgery(s) for that trauma will be excluded. Any operation performed after the patient has been discharged from the trauma stay will be included.
-Transplant cases - specifically: A patient who is admitted to the hospital for a transplant and has a transplant procedure and any additional surgical procedure during the transplant hospitalization will be excluded. Any operation performed after the patient has been discharged from the transplant stay will be included.
- American Society of Anesthesiologists score 6 (brain-death organ donors).
-Concurrent case - an additional operative procedure performed by a different surgical team under the same anesthetic (for example, coronary artery bypass graft procedure on a patient who is also undergoing a carotid endarterectomy). An assessment is not required on the concurrent procedure; however, this procedure would be reported as 'concurrent' in the operative section for the assessed case.

To ensure a diverse surgical case mix, also excluded (at each centre) are:

-More than 3 inguinal herniorrhaphies in an 8-day period.
-More than 3 breast lumpectomies in an 8-day period.
-More than 3 laparoscopic cholecystectomies in an 8-day period.
-If the site is collecting Urology cases, more than 3 transurethral resection of the prostate and/or transurethral resection of bladder tumor in an 8-day period.

Abbreviations: ACS NSQIP, American College of Surgeons National Surgical Quality Improvement Program; CPT, Current Procedural Terminology.
